# A differentiated approach to the choice of a neuroprotective drugs during complex antidepressant therapy of elderly depressive patients in a hospital setting

**DOI:** 10.1192/j.eurpsy.2024.434

**Published:** 2024-08-27

**Authors:** V. Pochueva, O. Yakovleva, T. Safarova

**Affiliations:** FSBSI “Mental Health Research Center”, Moscow, Russian Federation

## Abstract

**Introduction:**

The complex antidepressant therapy in combination with neuroprotectors increases the overall effectiveness of the treatment of depression in the elderly due to the group of the most difficult patients for therapy with ≥2 predictors of a low therapeutic response (LTRP), as well as in patients with complex (anxious, senesto-hypochondriacal, delusional) and prolonged (≥6 months) depressions.

**Objectives:**

Development of a differentiated approach to the choice of types of neuroprotective drugs in the course of complex antidepressant therapy in depressive elderly patients.

**Methods:**

We studied groups of hospitalized patients aged ≥60 years with mild, moderate and severe depression (according to ICD-10) who received antidepressant monotherapy (comparison group) for 28 days (43 people) or complex antidepressant therapy in combination with carnicetin (20), cerebrolysin (20), citicoline (20), ethylmethylhydroxypyridine succinate (EMHPS) (25) and actovegin (25). Complaints about memory impairment, lonely living, and the presence of leukoaraiosis on brain CT were considered as LTRP. Efficacy of therapy (change in total HAMD-17 scores in %) was compared in subgroups with neuroprotectors and in the comparison group in patients with ≥2 LTRPs, as well as in patients with complex and prolonged depressions. Statistical analysis was performed.

**Results:**

By the 28th day of treatment, all patients with ≥2 LTRPs in the subgroups with the addition of any neuroprotectors were responders (≥50% change) with a significantly higher efficacy of therapy than in the monotherapy group (36.0%, p<0.05). The efficacy of therapy was significantly higher in the subgroup with the addition of actovegin than in the subgroups with cerebrolysin and citicoline (73.7% versus 55.6% and 52.0%, respectively, p<0.05). In complex depression, the effectiveness of therapy in the subgroup with cerebrolysin did not statistically differ from the comparison group. In prolonged depression, no statistically significant difference in efficacy was found between the citicoline-supplemented subgroup and the monotherapy group. The highest efficacy in the treatment of complex and prolonged depression was observed in subgroups with the addition of actovegin and EMHPS (p<0.01).

**Conclusions:**

If there are indications for prescribing complex antidepressant therapy (≥2 LTRPs) in depressive patients of late age, all studied neuroprotective drugs demonstrated high efficiency compared to monotherapy. In complex depressions, the combination of antidepressants with cerebrolysin turned out to be less effective, in protracted depressions - with citicoline. Neuroprotectors actovegin and EMHPS can be considered universal drugs of choice for complex therapy of the most difficult categories of elderly depressive patients in a hospital setting.

**Disclosure of Interest:**

None Declared

